# Genetic basis of orange spot formation in the guppy (*Poecilia reticulata*)

**DOI:** 10.1186/s12862-021-01942-2

**Published:** 2021-11-25

**Authors:** Mayuko Kawamoto, Yuu Ishii, Masakado Kawata

**Affiliations:** Graduate School of Life Sciences, Tohoku University, 6-3, Aramaki Aza Aoba, Aoba-ku, Sendai, 980-8578 Japan

**Keywords:** Mate choice, Colored ornaments, Color-related genes, RNA-seq

## Abstract

**Background:**

To understand the evolutionary significance of female mate choice for colorful male ornamentation, the underlying regulatory mechanisms of such ornamentation must be understood for examining how the ornaments are associated with “male qualities” that increase the fitness or sexual attractiveness of offspring. In the guppy (*Poecilia reticulata*), an established model system for research on sexual selection, females prefer males possessing larger and more highly saturated orange spots as potential mates. Although previous studies have identified some chromosome regions and genes associated with orange spot formation, the regulation and involvement of these genetic elements in orange spot formation have not been elucidated. In this study, the expression patterns of genes specific to orange spots and certain color developmental stages were investigated using RNA-seq to reveal the genetic basis of orange spot formation.

**Results:**

Comparing the gene expression levels of male guppy skin with orange spots (orange skin) with those without any color spots (dull skin) from the same individuals identified 1102 differentially expressed genes (DEGs), including 630 upregulated genes and 472 downregulated genes in the orange skin. Additionally, the gene expression levels of the whole trunk skin were compared among the three developmental stages and 2247 genes were identified as DEGs according to color development. These analyses indicated that secondary differentiation of xanthophores may affect orange spot formation.

**Conclusions:**

The results suggested that orange spots might be formed by secondary differentiation, rather than de novo generation, of xanthophores, which is induced by Csf1 and thyroid hormone signaling pathways. Furthermore, we suggested candidate genes associated with the areas and saturation levels of orange spots, which are both believed to be important for female mate choice and independently regulated. This study provides insights into the genetic and cellular regulatory mechanisms underlying orange spot formation, which would help to elucidate how these processes are evolutionarily maintained as ornamental traits relevant to sexual selection.

**Supplementary Information:**

The online version contains supplementary material available at 10.1186/s12862-021-01942-2.

## Background

Colorful male ornamentation is important for female mate choice in many organisms. It has been proposed that colorful ornaments evolved as indicators of “male qualities” that increase the fitness (“good genes” models [1, 2]) or sexual attractiveness (Fisher’s runaway process [3]) of offspring. However, the mechanism underlying the relationships between the ornaments and “male qualities” remains unclear [4–7]. The guppy (*Poecilia reticulata*) is a useful model for studying sexual selection based on body coloration [4, 8]. Guppies are small live-bearers native to freshwater streams in Trinidad and South America [9]. As a typical example of sexual dimorphism, male guppies have vivid color spots and are believed to be the most color-polymorphic vertebrates [8]. Females choose their mates based on various characteristics and tend to prefer males with larger and more highly saturated orange spots [10, 11].

Previous studies have suggested that female guppies can recognize and evaluate orange spots based on the relative area and color saturation, as potential indicators of “male qualities” [5]. The color saturation of orange spots is determined by carotenoid uptake [11] and is also affected by parasite load [12], implying that the saturation is reflective of male health. Additionally, the area of orange spots has been correlated with sperm competitiveness and the ability of offspring to avoid capture [13, 14], suggesting that orange spots may be an indicator of offspring fitness (“good genes” models) [1, 2]. It is also believed that orange spots are an indicator of offspring attractiveness, as described by the “Fisher’s runaway process” [3], since the area of orange spots is heritable and at least partially genetically controlled [15–17].

Morphologically, the color spots of the guppy are organized by pigment cells, which are derived from neural crest cells (NCCs) [18, 19]. At least three types of pigment cells have been characterized in the guppy skin: black melanophores, yellow to orange xanthophores, and iridescent iridophores. Orange spots are formed mainly by xanthophores and iridophores [18]. Xanthophores retain pteridines and carotenoids, which are yellow, orange to red pigments, and configure the coloration of orange spots [20]. Pteridines are synthesized de novo by lysosome-like organelles called pterinosomes [20, 21], whereas carotenoids are obtained from food sources, such as unicellular algae [20].

Despite a long history of experimental and theoretical studies, the genetic basis underlying the formation of orange spots in male guppies, which is required to identify the mechanisms underlying the relationships between orange spots and “male qualities,” remains unclear. Previous crossing experiments suggest that many genes related to color patterns are sex-linked (reviewed in [22]). Meanwhile, quantitative trait loci analysis correlated several autosomal regions with the area of orange spots [23]. A genetic study indicated that a guppy mutant line called *blue*, which possesses a mutation in *colony-stimulating factor 1 receptor a* (*csf1ra*) gene, had no orange spots because of defective xanthophore formation [24]. Although these studies have successfully linked some chromosome regions and genes to orange spot formation, the regulation and roles of these genetic elements in the development of orange spots have not yet been elucidated.

In this study, the expression levels of genes in the male guppy skin were subjected to RNA-seq to reveal the genetic basis of orange spot formation. Three developmental stages were defined according to the color development in male guppies. Then, the gene expression levels were compared in two ways. First, the gene expression levels of male guppy skin with orange spots (orange skin) were compared with those without any color spots (dull skin) from the same individuals to identify the gene expression patterns specific for orange skin. Second, the gene expression levels of the whole trunk skin were compared among the three developmental stages to detect genes that change expression levels according to color development. Based on gene ontology enrichment analysis and pigment cell-related gene analysis of differentially expressed genes (DEGs), a model for the genetic basis of orange spot formation was proposed.

## Results

### Observations of color development

**Fig. 1:**
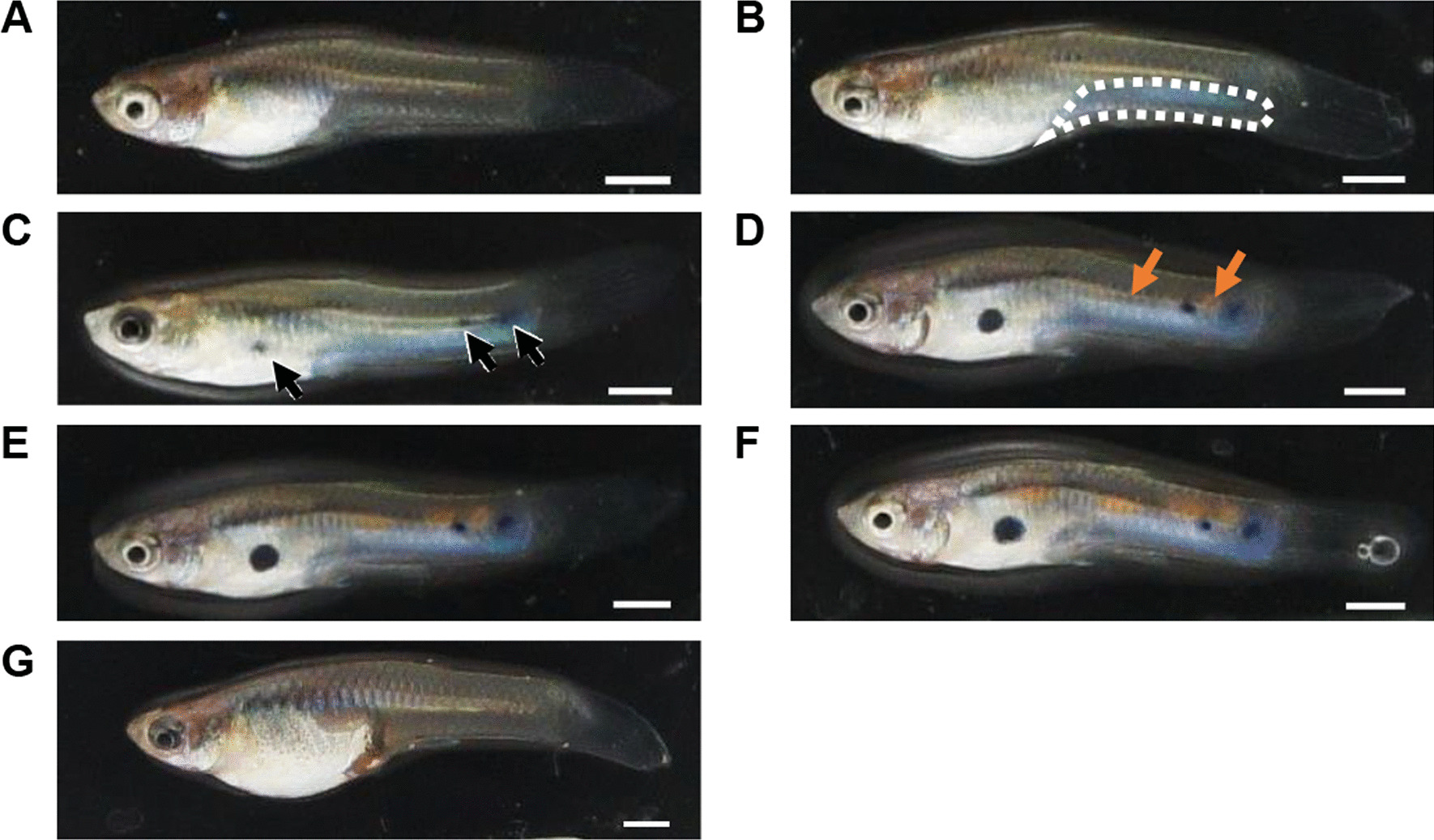
The body color of the guppy. Typical color development of a male guppy at **A** 54 days after birth. Iridescent coloration started to develop (enclosed by a white dotted line) at **B** 75 days after birth. Black spots started to develop (black arrows) at **C** 85 days after birth. Orange spots started to develop (orange arrows) at **D** 99 days after birth and enlarged at **E** 106 days after birth. Development of body coloration was completed at **F** 120 days after birth. **G** Body color of a mature female guppy at 127 days after birth. Scale bars: 2 mm

**Fig. 2:**
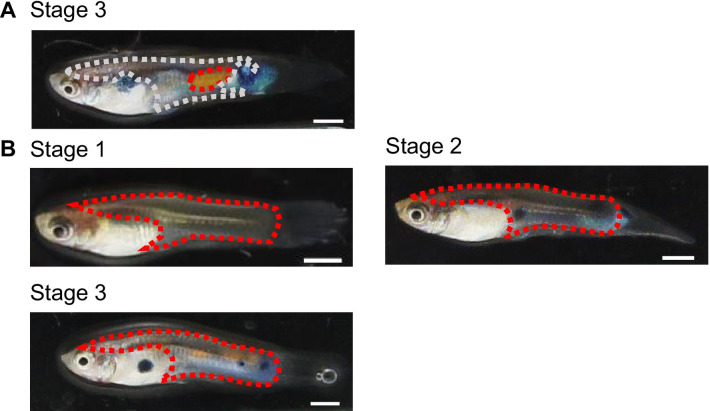
Examples of skin regions used as RNA-seq samples. RNA was extracted from each area enclosed by dotted lines. **A** Regions of the orange skin (enclosed by a red dotted line) and the dull skin (enclosed by a gray dotted line). **B** Regions used as the whole trunk skin (enclosed by red dotted lines). Scale bars: 2 mm

To determine how body color develops in male guppies, changes in the body colors of individuals were recorded from the start of gonopodia formation until the completion of body coloration (Fig. 1). Morphologically, males appeared almost the same as females at the start of gonopodia formation (Fig. 1a, g). Then, an iridescence color developed in males (Fig. 1b), followed by the formation of black spots (Fig. 1c) and finally orange spots (Fig. 1d), which initially appeared as lines on the central line and gradually enlarged (Fig. 1e, f). The following criteria were used to differentiate the three stages of color development for later experiments: stage 1 was defined as the period from the start of gonopodia formation before the emergence of male-specific body coloration, whereas stage 2 was defined as the start of orange spot formation, and stage 3 was defined as the completion of body coloration at approximately 3–4 weeks after stage 2 (Fig. 2, Additional file 1).

### DEGs

From 0.1–3.63 μg of total RNA, 41,076,226–60,359,952 raw RNA-seq reads were obtained (Additional file 2). No significant difference was observed in the number of read counts between samples; therefore, all sequence results were used for later analyses as they were not expected to affect the results. After quality control of raw RNA-seq reads, 12,858,878–21,059,959 reads were aligned to the female guppy genome (Additional file 2). Comparison of orange/dull skin within individuals (Fig. 2a, Additional file 1a) identified 1102 DEGs (Additional file 3), including 630 significantly upregulated and 472 downregulated in the orange skin as compared with the dull skin from the same individuals. Stage comparisons (Fig. 2b, Additional file 1b) identified 2247 DEGs (Additional file 4). Gene ontology (GO) enrichment analysis of the DEGs upregulated in the orange skin specimens resulted in four enriched GO terms (Table 1, Additional file 5).

**Table 1:** The result of gene ontology (GO) enrichment analysis

GO term accession	GO term name	FDR
GO:0006189	‘de novo’ IMP biosynthetic process	0.000296
GO:0006164	Purine nucleotide biosynthetic process	0.000545
GO:0043473	Pigmentation	0.030776
GO:0016192	Vesicle-mediated transport	0.042623

GO enrichment analysis of the differentially expressed genes (DEGs) that were upregulated in the orange skin specimens was resulted in four enriched GO terms. The enrichment criterion was a false discovery rate (FDR) of < 0.05

### Xanthophore-related genes

Xanthophores are one of the two types of pigment cells that mainly form orange spots [18]. Thus, the xanthophore-related genes identified in previous studies (Additional file 6) were classified by associations to xanthophore development, pteridine synthesis, and carotenoids as xanthophore-related genes (Additional file 6). Of the genes involved in xanthophore development, six genes were upregulated in the orange skin specimens, including four genes known to affect other types of pigment cells (Table 2). Two xanthophore development-related genes were identified by the stage comparison. The expression levels of *pax3b* decreased with color development, whereas *thra*, also affecting melanophores [25], was highest in stage 3 (Table 3). Of the genes related to pteridine synthesis, five were upregulated in the orange skin specimens, including four genes that also function in other types of pigment cells than xanthophores (Table 2). *gch2* was also detected by the stage comparison and the expression level was highest in stage 2 (Table 3). Of the genes related to carotenoids, four were upregulated in the orange skin specimens (Table 2). Stage comparisons identified two carotenoid-related DEGs and their expression levels were highest in stage 2 (Table 3).

**Table 2:** Xanthophore and iridophore-related DEGs detected by comparisons of the orange and dull skin specimens

Pigmentary function	Gene name	logFC	FDR
Xanthophore			
Xanthophore development	*csf1b (LOC103467541)*	1.56187733	7.53E−21
	csf1ra*	1.63430688	2.96E−24
	ednrba (LOC103478941)*	1.24385446	5.81E−10
	*pax7a*	2.58940395	1.20E−61
	sox10*	0.75461751	0.00019484
	thra (LOC103468293)*	0.8015877	1.46E−05
Pteridine synthesis	gart*	0.98145905	2.40E−12
	gch2 (LOC103481546)*	3.3024431	3.57E−41
	paics (LOC103463823)*	1.17429464	3.37E−15
	pts*	0.91297172	3.58E−08
	*xdh*	1.47551965	0.00067471
Carotenoid related	*bscl2 (LOC103471440)*	2.47443216	1.88E−23
	*plin6 (LOC103477535)*	2.23792664	7.62E−13
	*scarb1*	0.71637304	9.37E−06
	*ttc39bl (LOC103472854)*	2.38355104	6.61E−13
Iridophore			
Iridophore development	ednrba (LOC103478941)*	1.24385446	5.81E−10
	*fhl2l (LOC103457533)*	2.5165114	5.51E−14
	gpnmb*	2.87475033	7.17E−19
	*ltk (LOC103458875)*	1.20535128	1.62E−08
	*mpv17*	0.57722503	0.00433149
	sox10*	0.75461751	0.00019484
	*tfec*	1.60957677	6.55E−08
Guanine synthesis	gart*	0.98145905	2.40E−12
	paics (LOC103463823)*	1.17429464	3.37E−15
	*pnp4a (LOC103464790)*	2.48670961	1.74E−14

Asterisks indicate genes with known effects on multiple pigment cells (xanthophore, iridophore, and melanophore). Upregulation and downregulation of genes in the orange skin specimens are presented as positive and negative log2-fold change (logFC) values, respectively

**Table 3:** Xanthophore and iridophore-related genes extracted from DEGs detected by stage comparison

Pigmentary function	Gene name	logFC stage2	logFC stage 3	FDR
Xanthophore				
Xanthophore development	*pax3b*	− 0.240074049	− 1.024506408	0.00168639
	thra (LOC103468293)*	0.8422285	0.984864278	0.00010455
Pteridine synthesis	gch2 (LOC103481546)*	2.596284167	1.346388342	5.17E−05
Carotenoid related	*bscl2 (LOC103471440)*	1.760755604	0.88253586	0.00109993
	*scarb1*	0.942303034	0.368419924	0.00675451
Iridophore				
Iridophore development	edn3 (LOC103465492)*	− 1.219667499	− 1.091538668	8.62E−05
	*fhl2a*	1.544710776	1.561272758	5.95E−06
	*fhl2l (LOC103457533)*	3.662766117	3.20685195	7.18E−18
	gpnmb*	2.676380573	2.196925532	1.11E−17
	tfap2a*	0.390339609	− 0.532566067	0.00150917
	*tfec*	1.746850047	0.941123134	8.82E−06
	vps18*	0.380195963	− 0.392171768	0.00976626
Guanine synthesis	*pnp4a (LOC103464790)*	3.628065427	2.305069974	8.31E−10

Asterisks indicate genes with known effects on multiple pigment cells (xanthophore, iridophore, and melanophore). Upregulation and downregulation of genes in the stage 2 and 3 compared to stage 1 are presented as positive and negative logFC (logFC stage2, logFC stage3) values, respectively

### Iridophore-related genes

Another type of pigment cell that forms orange spots is iridophore, which contains guanine crystals that produce structural color [18]. Genes involved in iridophore development and guanine synthesis were included as iridophore-related genes (Additional file 6). Of the iridophore development-related genes, seven were upregulated in the orange skin specimens, and three of them are also involved in the development of other types of pigment cells (Table 2). Stage comparison identified seven DEGs, containing four genes that function in multiple types of pigment cells, with the highest expression in stage 2, except for *edn3* and *fhl2a*, most highly expressed in stage 1 and stage 3, respectively (Table 3). Three genes associated with guanine synthesis were upregulated in the orange skin specimens, including two genes also associated with other types of pigment cells (Table 2). Among those, *pnp4a* was also detected by the stage comparison and most highly expressed in stage 2 (Table 3).

## Discussion

In this study, the expression levels of genes in male guppy skin were investigated to understand the genetic basis of orange spot formation. In the orange skin specimens, 630 DEGs were upregulated and 472 were downregulated (Additional file 3). Additionally, 2247 DEGs were identified among the three color-developmental stages (Additional file 4).

### Pigmentation-related genes contribute to orange spot formation

GO enrichment analysis of DEGs upregulated in the orange skin specimens detected four enriched GO terms (Table 1), all of which could be related to pigmentation. DEGs with the “pigmentation” term are known to function in the development of pigment cells, pigment synthesis, and melanosomes (Additional file 5) [26–30]. Pteridine and guanine are synthesized from inosine monophosphate (IMP), a type of purine nucleotide, which is also required for melanin synthesis [28]. For vesicle-mediated transport, melanosomes, which synthesize and transport melanin in melanophores, are formed by the fusion of vesicles from the Golgi complex and endoplasmic reticulum [21]. Pteridine and guanine crystals are believed to be synthesized by organelles similar to melanosomes but distinctively defined as pterinosomes and iridosomes in pigment cells, respectively [31]. Considering orange spots are formed mainly by xanthophores and iridophores [18], DEGs with enriched GO terms may contribute to orange spot formation by involving the development of the two types of pigment cells and in the synthesis and transport of pigments in these cells.

### Xanthophore differentiation during orange spot development

**Fig. 3:**
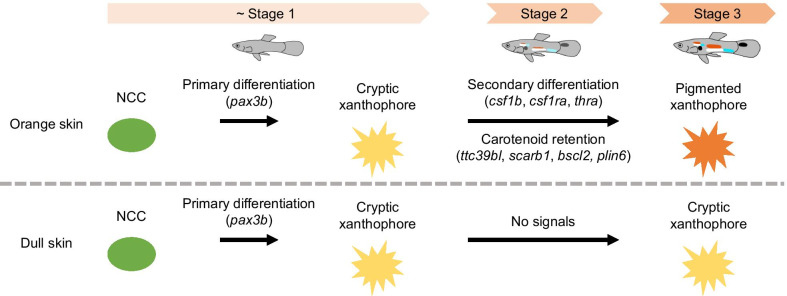
A schematic model of orange spot formation. Cryptic xanthophores differentiate from NCCs and cover the whole body until male-specific coloration begins to develop. In orange skin, cryptic xanthophores that respond to Csf1 and TH signaling differentiate into pigmented xanthophores, increase carotenoid retention, and form orange spots. In dull skin, cryptic xanthophores do not perceive signals inducing secondary differentiation, thus orange spots are not formed

Xanthophore- and iridophore-related genes were detected as DEGs, suggesting that genes involved in secondary differentiation of xanthophores play a major role in orange spot formation (Fig. 3). The expression levels of genes involved in NCC migration and specification (i.e., *pax7a*, *ednrba*, and *sox10*) [26, 32, 33] were comparatively upregulated in the orange skin specimens (Table 2). However, *pax7a* is also associated with pterinosome diffusion [34], whereas *ednrba* and *sox10* are commonly expressed in precursors of xanthophores, iridophores, and melanophores [35, 36]. Meanwhile, the expression levels of *sox10* and *ednrba* are maintained in differentiated iridophores [32, 37]. For the stage comparison, the expression levels of *tfap2a* and *tfec*, which have been implicated in the specification of iridophores from NCCs [29, 38], were highest in stage 2 (Table 3), which seemed to be consistent with color development (Figs. 1 and 2). Conversely, the expression level of *pax3b*, which is involved in the specification of xanthophores from NCCs [34], decreased with color development (Table 3). Thus, the expression patterns of these genes do not necessarily indicate de novo primary differentiation of xanthophores from NCCs in orange skin.

In zebrafish, yellow interstripes are formed by region-specific differentiation in which cryptic larval xanthophores are differentiated into adult pigmented xanthophores (secondary differentiation) [39]. The secondary differentiation of xanthophores is induced by two signals, namely, Csf1 and thyroid hormone (TH) signaling [39, 40]. In the orange skin specimens, both the ligand (*csf1b*) and receptor gene (*csf1ra*) of Csf1 signaling were upregulated (Table 2). Regarding TH signaling, genes encoding the TH receptor (*thra*) were upregulated in the orange skin specimens (Table 2). Additionally, the expression levels of *thra* were found to increase with color development (Table 3). These results suggest that Csf1 and TH signaling affect orange spot formation in adult male guppies.

Changes in the expression levels of pteridine and carotenoid-related genes also support the role of secondary differentiation of xanthophores in orange spot development. Genes related to pteridine synthesis (*gart*, *gch2*, *paics*, *pts*, *xdh*) [28, 41–43], carotenoid uptake (*ttc39bl*, *scarb1*) [25, 44], and carotenoid accumulation (*bscl2*, *plin6*) [25, 45] were upregulated in the orange skin specimens. The expression levels of *gch2*, *bscl2*, and *scarb1* also changed with color development and were highest in stage 2 (Table 3). In zebrafish, the expression levels of carotenoid retention-related genes are higher in adult xanthophores than larval xanthophores, which is believed to cause the color change of xanthophores due to secondary differentiation, whereas those of pteridine synthesis-related genes were more similar across stages of xanthophore development [25]. Like the adult xanthophores of zebrafish, the expression pattern of carotenoid-related genes, especially *bscl2* and *scarb1*, may indicate an importance of the secondary differentiation of xanthophores in orange spot formation. Taken together, in adult guppies, secondary differentiation of xanthophores that respond to Csf1 and TH signals is likely to affect orange spot formation more than primary differentiation of xanthophores from NCCs (Fig. 3).

However, further experiments will be needed to clarify the contribution of secondary differentiation to orange spot formation. This RNA-seq analysis alone cannot determine whether the differences in gene expression levels detected in the two comparisons are caused by differences in the number of cells, differences within cells, or both. Furthermore, it is unclear as to which types of cells were affected by the DEGs detected by the stage comparison, especially genes with multiple functions (Indicated by asterisks in Table 3). It is necessary to investigate the two types of xanthophores on the male skin and the changes in their distribution during color development, as well as the effects of artificial modulation of the Csf1 and TH signaling on xanthophores.

### Candidate genes regulating the areas and saturation levels of orange spots

The results of this study indicate that orange spots are formed by secondary differentiation of xanthophores. This suggests that genes regulating the secondary differentiation of xanthophores could be associated with the area and saturation of orange spots, which are essential traits in female mate choice [10, 11]. Previous studies indicate that the areas of orange spots are heritable among males; thus, genetic variation influences these areas [15, 16]. The results of the present study suggest that secondary differentiation of larval cryptic xanthophores into adult pigmented xanthophores occurs during orange spot formation, which is induced by Csf1 and TH signaling [39, 40] (Fig. 3), and genes involved in these signaling pathways might include a regulator of the areas of orange spots functioning in female mate choice.

In particular, Csf1 signaling is expected to be key in the induction of region-specific secondary differentiation of xanthophores. In zebrafish, the Csf1 ligand gene (*csf1*) is expressed by iridophores [46], whereas the receptor gene (*csf1r*) is expressed by xanthophores [43]. Iridophores emerge in the skin from the horizontal myoseptum, and the first yellow stripe is formed in the iridophore-rich area where xanthophore secondary differentiation is initiated [40, 46]. Csf1 signaling is also suggested to be involved in the development of anal fin egg-spots in cichlids [47, 48] and red pectoral fins in a species of medakas (*Oryzias woworae*) [49]. A role of Csf1 signaling in the secondary differentiation of xanthophores for body color formation might be common in most fish. However, there was no study showing this possibility except for the zebrafish. For orange spots in male guppies, Kottler et al. [18] showed that these spots are formed by xanthophores and iridophores. The results of the present study showed that most orange spots were formed approximately on the horizontal myoseptum (Fig. 1, Additional file 1) and both the ligand (*csf1b*) and receptor gene (*csf1ra*) of Csf1 signaling were upregulated in the orange skin specimens (Table 2), suggesting that a mechanism similar to that in zebrafish could regulate the areas of orange spots on the skin of the male guppy. In addition, *choker* mutants in zebrafish that lack the horizontal myoseptum have a meandering striped pattern due to the absence of iridophore migration through the horizontal myoseptum [50]. Thus, it is expected that muscle development will also constrain the region where secondary differentiation of xanthophore occurs and the position of the orange spot. Examining the relationships among the positions of orange spots, xanthophores, iridophores, and Csf1 ligands concerning morphogenesis will elucidate how the areas of orange spots are regulated at the genetic and cellular levels and maintained as a heritable trait. This contributes to understanding the evolution of mate choice based on orange spots through the Fisher’s runaway process [3] and/or “good genes” models [1, 2].

Additionally, investigations of the effects of Csf1 signaling may provide genetic evidence for “good genes” models [1, 2] of the evolution of orange spots as an indicator of “male quality”. It is known that *csf1r* affects the development of tissue-resident macrophages, brain microglia, Langerhans cells, Paneth cells, and osteoclasts as well as pigment cells [51–55], indicating that Csf1 signaling plays important roles in immunocompetency and skeletal development as well as orange spot formation. Hence, genes related to Csf1 signaling could include “good genes” associated with colorful ornamentation as an indicator of offspring fitness.

Besides areas of orange spots, the color saturation of orange spots is an essential factor in female mate choice, and our data provide a model for the genetic basis of this trait. Although color saturation of orange spots is reportedly affected by the level of carotenoids in the diet and parasite load [11, 12], the regulatory mechanism of color saturation is unclear. It has been hypothesized that body color due to carotenoids is not a direct reflection of the amount of carotenoids ingested but rather an indicator of normal cellular processes regulated by retinoids, which are produced from carotenoids [56]. As discussed earlier, our data suggest that color change and maintenance through xanthophore secondary differentiation are associated with carotenoid retention-related genes (i.e., *ttc39bl, scarb1, bscl2, plin6*) (Fig. 3, Tables 2 and 3). Overall, carotenoid intake may enhance orange saturation through the activation of carotenoid retention-related genes. Hence, further investigations of the potential effect of carotenoid intake on the activation of carotenoid retention-related genes may reveal the genetic basis of the relationship between color saturation of orange spots and foraging ability or parasite infection, as well as “good genes” associated with the health of offspring.

We note that the RNA-seq reads were aligned to the female reference genome due to the lack of a complete male-specific Y unique sequence [57]. Considering that females also have the same types of pigment cells as males [24], it is likely that many genes expressed in the male skin are similar to those of females. However, since areas of orange spots are inherited in males [15, 16], it is possible that further studies that focus on male-specific Y-linked genes, not analyzed in this study, may elucidate important roles of such genes in orange spot formation.

## Conclusions

In this study, we compared gene expression levels in male guppy skins and detected gene expression patterns specific to orange spots and certain color developmental stages (Additional files 3 and 4). The GO enrichment analysis showed that genes involved in the development of pigment cells, pigment synthesis, and transport are upregulated in the orange skin specimens and these genes may affect orange spot formation via xanthophore and iridophore (Table 1, Additional file 5). The expression patterns of genes related to xanthophore and iridophore suggest that orange spots are formed by secondary differentiation of xanthophores mediated by the Csf1 and TH signaling pathways (Fig. 3, Tables 2 and 3). Overall, our data provided the first, well-annotated list of candidate genes related to the area and saturation of orange spots in male guppies, which helps explain the genetic basis of body color development critical for sexual selection. This provides insight into how orange spots are genetically linked with “male quality” and will contribute to further understanding of the evolutionary process of sexual selection based on ornamental traits.

## Methods

### Animal collection and maintenance

Female guppies were collected in 2019 from a long-established feral population in Okinawa Prefectural Plant Protection Center (Okinawa, Japan). Okinawa guppy populations were introduced and established more than 60 years ago from escaped or thrown pet guppies [58]. The females that mated in the wild before capture and became pregnant were selected, and their offspring were obtained in the laboratory. Permission to collect guppies was obtained from Okinawa Prefectural Plant Protection Center in advance. The offspring were reared under a 12 h light/dark cycle at 25 ℃ ± 1 ℃. All fish were fed flake food for ornamental fish (TetraMin; Spectrum Brands Pet, LLC, Blacksburg, VA, USA) 1–2 times/day. The animal care and breeding protocols were performed following the guidelines of the Animal Care and Use Committee of Tohoku University (Sendai, Japan; permit number: 2017Seidou-025).

### Observation of color development

Color development of male guppies was recorded approximately once per week starting from gonopodia formation at the age of 2–3 months. Guppies were anesthetized with 0.05% 2-phenoxyethanol, transferred to a petri dish, photographed with a camera (NEX-5; Sony Corporation, Tokyo, Japan) equipped with an alpha lens (SEL18200LE; Sony Corporation).

### Sampling of skin tissue and RNA extraction

After being photographed, guppies were classified as one of three developmental stages, rapidly decapitated, then soaked in RNA*later* solution (Thermo Fisher Scientific, Waltham, MA, USA) and stored at − 30 ℃. One male was randomly chosen from the offspring of each pregnant female caught in the field. After thawing, the orange skin (with orange spots) and dull skin (lacking any color spots) were collected separately using scissors and forceps from half of the same individuals classified as developmental stage 3 (Fig. 2a, Additional file 1a). For individuals classified as developmental stages 1 and 2 and the remaining half of individuals classified as developmental stage 3, the skin of the whole trunk, except for the abdomen, was collected (Fig. 2b, Additional file 1b). Total RNA was extracted from the skin tissue using TRIzol Reagent (Invitrogen Corporation, Carlsbad, CA, USA). A single RNA sample was extracted from the skin of one individual. The RNA concentration and purity were measured with a Nanodrop spectrophotometer (NanoDrop Technologies, LLC, Wilmington, DE, USA) and Agilent Bioanalyzer (Agilent Technologies, Inc., Santa Clara, CA, USA).

### RNA-seq and gene expression analysis

RNA-seq analysis was performed of the total RNA samples extracted from the orange skin, dull skin, and whole trunk skin specimens of guppies at developmental stages 1–3. Five biological replicates were prepared for each condition. Library preparation and sequencing were outsourced to Macrogen Japan (Tokyo, Japan). A library was generated using a TruSeq Stranded mRNA Library Prep Kit (Illumina, Inc., San Diego, CA, USA). Sequencing was performed with a NovaSeq 6000 System (Illumina, Inc.) and 100-bp paired-end reads were generated (Additional file 2). Quality control of raw RNA-seq reads was conducted with the prinseq++1.2 tool [59]. Reads were trimmed recursively from the 3′-end chunks of sequences 2 bp in length if the mean quality score of the first five bases was < 30 and removed if shorter than 36 bp or with a mean quality score of < 25 (Additional file 2). The RSEM 1.3.1 software package [60] and STAR 2.7.3 algorithm [61] with default parameters were used to align reads with the open reading frames of the guppy genome (RefSeq assembly accession: GCF_000633615.1) and to calculate gene expression levels. DEGs were identified using the edgeR 3.28.0 bioconductor package [62]. Gene expression levels were compared between the orange and dull skin corresponding to skin collected from the same individuals (orange/dull skin comparison) (Additional file 3) and among the whole trunk skin samples of individuals classified as developmental stages 1–3 (stage comparison) (Additional file 4). Genes with a false discovery rate (FDR) of < 0.01 were considered DEGs. GO (biological process) enrichment analysis of DEGs that were upregulated in the orange skin specimens was conducted with the goatools 1.0.5 package [63] and GO terms of the guppy (Ensembl 104). The enrichment criterion was an FDR of < 0.05.

### Xanthophore and iridophore-related gene analysis

Genes known to be involved in xanthophore and iridophore were listed based on previous studies(Additional file 6) and extracted from the DEGs (Tables 2 and 3). The genes were subdivided into five groups: xanthophore development, pteridine synthesis, carotenoid related, iridophore, development, and guanine synthesis. Genes with known effects on multiple types of pigment cells were indicated with asterisks in Tables 2 and 3 and Additional file 6.

## Supplementary Information

**Additional file 1. **Body color of guppies used for RNA extraction. a Guppies used for the orange/dull skin comparison. b Guppies used for the stage comparison. Scale bars: 2 mm.

**Additional file 2. **Results of total RNA amount used for library generation, sequencing, and mapping of each sample.

**Additional file 3. **List of differentially expressed genes (DEGs) detected by comparison of the orange and dull skin specimens. Upregulation and downregulation of genes in the orange skin specimens are presented as positive and negative log2-fold change (logFC) values, respectively.

**Additional file 4. **List of DEGs detected by stage comparison. Upregulation and downregulation of genes in stages 2 and 3 compared to stage 1 are presented as positive and negative logFC (logFC stage2, logFC stage3) values, respectively.

**Additional file 5. **List of DEGs with enriched GO terms.

**Additional file 6. **List of xanthophore and iridophore-related genes. Asterisks indicate genes with known effects on multiple pigment cells (xanthophore, iridophore, and melanophore).

## Data Availability

The open reading frames of the guppy genome are available from NCBI (https://www.ncbi.nlm.nih.gov) under the RefSeq assembly accession number GCF_000633615.1. GO terms of the guppy are available from Ensembl (http://www.ensembl.org/) with Biomart option. RNA-seq raw sequence reads are available through the DDBJ Sequence Read Archive (https://ddbj.nig.ac.jp) under Accession no. DRA011617. All the other data are included in the Additional files. Funding M.K. was supported by a Grant-in-Aid for Scientific Research (18H02505 and 21H02558) from the Japan Society for the Promotion of Science.

## Abbreviations

bscl2: BSCL2 lipid droplet biogenesis associated, seipin
csf1(b): Colony stimulating factor 1(b)
csf1r(a): Colony-stimulating factor 1 receptor (a)
DEG: Differentially expressed gene
edn3: Endothelin-3
ednrba: Endothelin receptor Ba
FDR: False discovery rate
fhl2a: Four and a half LIM domains 2a
gart: Phosphoribosylglycinamide formyltransferase
gch2: GTP cyclohydrolase 2
GO: Gene ontology
IMP: Inosine monophosphate
logFC: Log2-fold change
NCC: Neural crest cell
paics: Phosphoribosylaminoimidazole carboxylase, Phosphoribosylaminoimidazole succinocarboxamide synthetase
pax3b: Paired box 3b
plin6: Perilipin 6
pnp4a: Purine nucleoside phosphorylase 4a
pts: 6-Pyruvoyltetrahydropterin synthase
scarb1: Scavenger receptor class B, member 1
sox10: SRY-box transcription factor 10
tfap2a: Transcription factor AP-2 alpha
tfec: Transcription factor EC
TH: Thyroid hormone
thra: Thyroid hormone receptor alpha
ttc39bl: Tetratricopeptide repeat protein 39B-like
xdh: Xanthine dehydrogenase
