# Supplementary figures and images for: Genetic basis of orange spot formation in the guppy (Poecilia reticulata)
Source: BMC Ecol Evol. 2021 Nov 25;21:211. doi: 10.1186/s12862-021-01942-2 (PMC8613973; doi:10.1186/s12862-021-01942-2)

## A Stage 3

0625-2

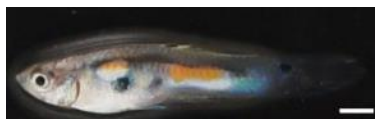

0627-2

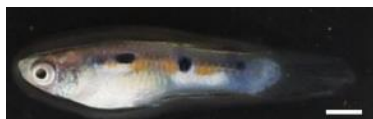

0628-3

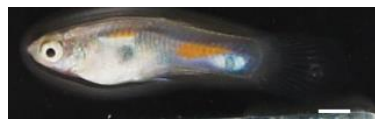

0707-3

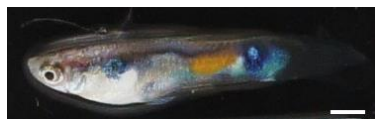

0728-1

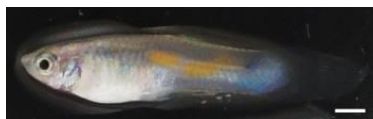

### B Stage 1

0629-1

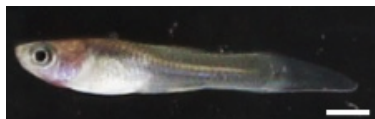

0707-1

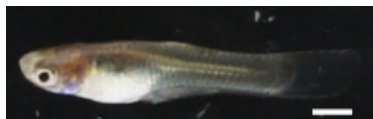

0710-1

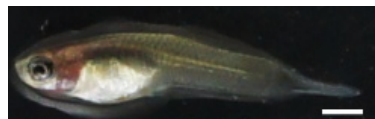

0718-2

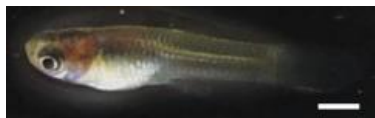

0813-1

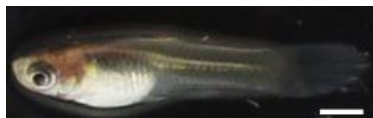

## Stage 2

0625-1

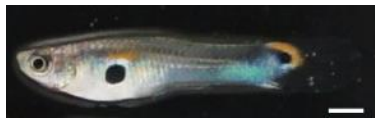

0627-3

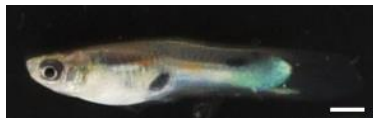

0628-2

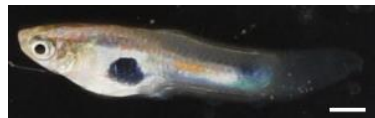

0712-1

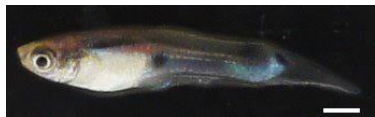

0821-1

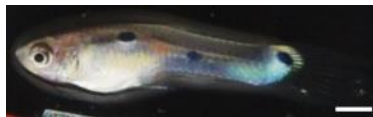

### Stage 3

0627-1

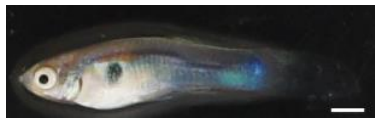

0707-4

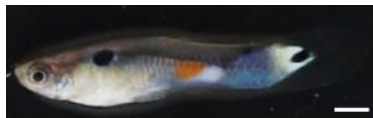

0709-2

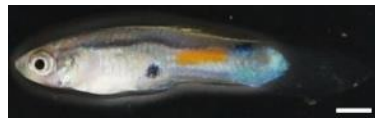

0710-2

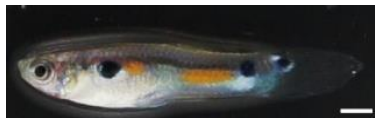

0718-1

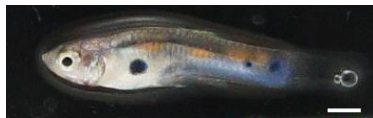

Supplement: Supplementary file 1 — Additional file 1. Body color of guppies used for RNA extraction. a Guppies used for the orange/dull skin comparison. b Guppies used for the stage comparison. Scale bars: 2 mm. [file 12862_2021_1942_MOESM1_ESM.pdf]
